# Effects of subchronic dietary exposure to the engineered nanomaterials SiO_2_ and CeO_2_ in C57BL/6J and 5xFAD Alzheimer model mice

**DOI:** 10.1186/s12989-022-00461-2

**Published:** 2022-03-25

**Authors:** Adriana Sofranko, Tina Wahle, Julia Kolling, Harm J. Heusinkveld, Burkhard Stahlmecke, Martin Rosenbruch, Catrin Albrecht, Roel P. F. Schins

**Affiliations:** 1 IUF - Leibniz Research Institute for Environmental Medicine, Auf’m Hennekamp 50, 40225 Düsseldorf, Germany; 2 National Institute for Public Health and the Environment (RIVM), Bilthoven, The Netherlands; 3 Institute for Energy and Environmental Technology e.V. (IUTA), Duisburg, Germany; 4 Heinrich- Heine University, Düsseldorf, Germany; 5 Present Address: State Office for Consumer Protection Saxony-Anhalt, Stendal, Germany

**Keywords:** Amorphous silica, Nanoceria, Subchronic oral exposure study, Neurobehavioral testing, Neurotoxicity, Alzheimer’s disease

## Abstract

**Background:**

There is an increasing concern about the neurotoxicity of engineered nanomaterials (NMs). To investigate the effects of subchronic oral exposures to SiO_2_ and CeO_2_ NMs on Alzheimer’s disease (AD)-like pathology, 5xFAD transgenic mice and their C57BL/6J littermates were fed ad libitum for 3 or 14 weeks with control food pellets, or pellets dosed with these respective NMs at 0.1% or 1% (w/w). Behaviour effects were evaluated by X-maze, string suspension, balance beam and open field tests. Brains were analysed for plaque load, beta-amyloid peptide levels, markers of oxidative stress and neuroinflammation.

**Results:**

No marked behavioural impairments were observed in the mice exposed to SiO_2_ or CeO_2_ and neither treatment resulted in accelerated plaque formation, increased oxidative stress or inflammation. In contrast, the 5xFAD mice exposed to 1% CeO_2_ for 14 weeks showed significantly lower hippocampal Aβ plaque load and improved locomotor activity compared to the corresponding controls.

**Conclusions:**

The findings from the present study suggest that long-term oral exposure to SiO_2_ or CeO_2_ NMs has no neurotoxic and AD-promoting effects. The reduced plaque burden observed in the mice following dietary CeO_2_ exposure warrants further investigation to establish the underlying mechanism, given the easy applicability of this administration method.

**Supplementary Information:**

The online version contains supplementary material available at 10.1186/s12989-022-00461-2.

## Introduction

The development and steady introduction of new engineered nanomaterials (NMs) to the market has raised awareness about potential adverse health effects resulting from long-term exposures. The health risk concerns for NMs originated from inhalation toxicology studies that could substantiate the role of ultrafine particles in the epidemiological link between ambient air pollution exposure and cardiopulmonary diseases (reviewed by [1]). Likewise, the awareness about potential adverse effects of NMs on the central nervous system came from inhalation studies in more recent years. Neuroinflammatory, neurotoxicological and neurodegenerative effects observed by inhaled ultrafine particles and NMs in these toxicological studies provided experimental support to the growing number of epidemiological studies that showed associations between particulate air pollution exposure and neurological diseases [2, 3].

Specific concern has risen that long-term exposure to particulate air pollution could contribute to the pathogenesis of Alzheimer’s disease (AD) the most common neurodegenerative disease in the world [3–5]. A major neuropathological hallmark of AD is the generation of hydrophobic Amyloid-β peptide (Aβ) containing plaques resulting from the sequential proteolysis of the amyloid precursor protein (APP) by β- and γ-secretase enzymes (reviewed in [6, 7]). Although the exact mechanisms of initiation and progression of AD are still incompletely understood, it has been suggested that specific NMs may be involved due to their ability to disrupt Aβ homeostasis, resulting from reactive oxygen species generation (ROS) and oxidative stress, in similarity with other environmental factors like specific neurotoxic metals and some pesticides [3, 8, 9].

With the growing evidence for a role of inhaled nanoparticles in AD, there is also an increasing debate regarding the neurotoxicity and potential AD-promoting effects of ingested NMs. Indeed, neurotoxic effects in mice have been reported following oral exposure to NMs composed of silver [10–12], zinc oxide [13, 14], titanium dioxide [15] and iron oxide [16]. However, to the best of our knowledge it has not yet been investigated if long-term oral exposure to NMs can promote the development and progression of AD. Therefore, the main goal of our study was to address if subchronic oral exposure to NMs can accelerate hallmarks of Alzheimer-like pathology in mice. For this purpose, we selected synthetic amorphous SiO_2_ (SAS) and CeO_2_ NMs (“nanoceria”), representing two of the most widely used and investigated types of nanoparticles.

SiO_2_ is extensively used in chemistry, agriculture and consumer products, including cosmetics [17, 18]. In the food sector, it finds application as an anti-caking agent in powdered food products and is listed in Europe as a food additive E551 [19]. CeO_2_ NMs are used as well in various commercial and industrial applications, e.g., as a catalyst, an ultraviolet-filter [20] and as a fuel additive to improve combustion [21]. They are also increasingly promoted in agricultural applications [22, 23]. Although CeO_2_ NMs are not used as a food additive, accumulation in agricultural crops and trophic transfer have been reported [24, 25]. Furthermore, as an additive to diesel and gasoline fuels, CeO_2_ NMs could be inhaled following their emission with the vehicle exhaust [21, 26] and subsequently reach the gastrointestinal tract following mucociliary clearance and swallowing as previously shown for other ultrafine particles [27, 28]. Finally, because of the coexistence of Ce^3+^ and Ce^4+^ in nanosized CeO_2_ and its resulting unique redox-active properties, nanoceria has also received rapidly growing attention in biomedical and pharmaceutical applications [29, 30].

For our present investigations, the SiO_2_ and CeO_2_ NMs were incorporated into mouse feed pellets at 1% and 0.1% weight/weight (w/w) concentrations. The 1% dosing of the NMs in the pellets was selected on the basis of the amount of SiO_2_ that should not be exceeded in food applications, i.e. 2%, according to the US Food and Drug Administration [31]. While we selected the same doses for both types of NMs, it should be emphasized that for CeO_2_ the anticipated human exposures are likely much lower than for the food additive SiO_2_.

For the investigation of neurotoxicity and AD-like pathology, female heterozygous 5xFAD mice [32, 33] and their female nontransgenic C57BL/6J littermates were used. The 5xFAD mouse model is characterized by a steadily increasing amyloid deposition, starting from the age of 2 months and continuing to increase until at least after the age of 6 months [32]. Phenotype-dependent memory impairments and motor deficits can be observed in these mice from the age of 4–6 months [33]. Accordingly, at an age of 9 weeks, the mice were fed ad libitum﻿ during 3 or 14 weeks with the various NM-dosed or control pellets. In the 3rd and 14th week of exposure neurotoxicity was assessed by a series of behavioural tests, while specific effects on AD-like pathology were evaluated in the 5xFAD mice by evaluation of plaque load, Aβ-peptide levels and markers of oxidative stress and neuroinflammation. With the same mouse model, we previously demonstrated that inhalation exposure to diesel engine exhaust results in an accelerated formation of Aβ-plaques as well as motor function impairment [9]. In the present study, general toxicity beyond the brain was concurrently assessed by analysis of body weight gain as well as gross examinations, weight and histopathological analyses of specific organs.

## Results

### Body and organ weight changes

**Fig. 1:**
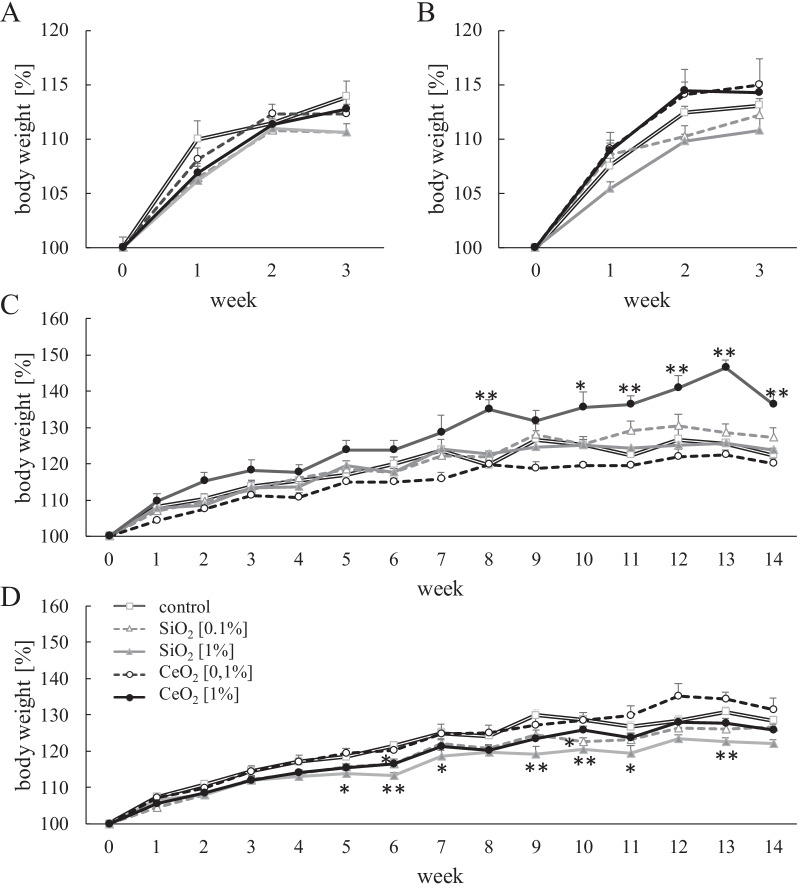
Body weight gain (%) of C57BL/6J (**A**, **C**) and 5xFAD (**B**, **D**) mice during 3 weeks (**A**, **B**) or 14 weeks (**C**, **D**) exposure to CeO_2_ or SiO_2_ [0.1% or 1%] in feed pellets ad libitum. Statistical analysis was performed using ANOVA followed by Dunnett evaluation; *p < 0.05 and **p ≤ 0.01 versus mice exposed to control feed pellets. Number of animals per group: 3 weeks C67BL/6J control (n = 6); SiO_2_ 0.1% (n = 6); SiO_2_ 1% (n = 7); CeO_2_ 0.1% (n = 6). CeO_2_ 1% (n = 6); 5xFAD control (n = 10); SiO_2_ 0.1% (n = 10); SiO_2_ 1% (n = 9); CeO_2_ 0.1% (n = 10). CeO_2_ 1% (n = 10); 14 weeks C67BL/6J control (n = 5); SiO_2_ 0.1% (n = 6); SiO_2_ 1% (n = 6); CeO_2_ 0.1% (n = 6). CeO_2_ 1% (n = 6); 5xFAD control (n = 11); SiO_2_ 0.1% (n = 9); SiO_2_ 1% (n = 10); CeO_2_ 0.1% (n = 10). CeO_2_ 1% (n = 10)

No effects on body weight gain were observed in the 5xFAD mice or their C57BL/6J littermates during 3 weeks of feeding with the SiO_2_ or CeO_2_ dosed feed pellets (see Fig. 1 and Table 1). In the 5xFAD mice that were exposed for 14 weeks to 1% SiO_2_, a reduction in body weight gain was observed from weeks 5–7 as well as from weeks 9–13 (Fig. 1D). In the corresponding non-transgenic mice, the subchronic exposure to SiO_2_ did not cause any significant reduction in body weight gain (Fig. 1C).

**Table 1:** Body and organ weights

	Control		SiO_2_ [0.1%]		SiO_2_ [1%]		CeO_2_ [0.1%]		CeO_2_ [1%]	
	C57BL/6J	5xFAD	C57BL/6J	5xFAD	C57BL/6J	5xFAD	C57BL/6J	5xFAD	C57BL/6J	5xFAD
*A*										
Body weight, sacrifice (g)	20.17 ± 1.32	19.15 ± 0.82	19.12 ± 1.03	18.78 ± 0.92	19.23 ± 0.67	18.58 ± 0.92	19.65 ± 1.51	19.23 ± 1.04	19.15 ± 0.91	19.20 ± 0.78
Body weight gain (g)^a^	2.47 ± 0.66	2.21 ± 0.33	1.80 ± 0.47	2.03 ± 0.61	1.83 ± 0.35	1.79 ± 1.03	2.17 ± 0.52	2.46 ± 1.17	2.15 ± 0.42	2.38 ± 0.47
Liver/body weight (mg g^−1^)	45.28 ± 8.89	44.71 ± 5.55	43.52 ± 4.78	47.36 ± 5.57	42.64 ± 6.70	45.27 ± 8.54	47.04 ± 2.06	44.50 ± 4.74	44.96 ± 3.04	44.08 ± 7.27
Spleen/body weight (mg g^−1^)	4.07 ± 0.31	3.80 ± 0.40	4.31 ± 0.76	4.10 ± 0.51	3.66 ± 0.47	4.23 ± 0.65	3.92 ± 0.66	3.92 ± 0.38	3.82 ± 0.29	3.89 ± 0.69
Kidney/body weight (mg g^−1^)	10.20 ± 3.01	8.99 ± 3.61	10.18 ± 3.22	8.60 ± 3.25	9.92 ± 2.62	8.60 ± 3.41	10.74 ± 3.82	8.60 ± 2.98	9.64 ± 2.83	9.22 ± 3.21
Colon/body weight (mg g^−1^)	8.19 ± 0.81	8.39 ± 1.37	8.19 ± 1.03	8.15 ± 1.09	8.17 ± 0.91	8.69 ± 0.85	8.55 ± 0.78	8.91 ± 0.78	8.03 ± 1.23	8.79 ± 1.22
Small int./body weight (mg g^−1^)	42.51 ± 6.00	42.38 ± 7.08	40.47 ± 6.60	46.40 ± 5.41	40.96 ± 7.20	40.89 ± 13.61	39.84 ± 7.16	44.34 ± 5.74	45.72 ± 1.55	46.69 ± 3.28
*B*										
Body weight, sacrifice (g)	21.68 ± 1.36	22.56 ± 1.25	23.72 ± 1.53	22.32 ± 2.24	22.33 ± 1.39	21.60 ± 1.03	22.47 ± 1.05	22.92 ± 1.65	25.20* ± 2.14	21.92 ± 0.26
Body weight gain (g)^a^	3.98 ± 0.80	4.96 ± 0.60	5.10 ± 1.19	4.72 ± 1.07	4.28 ± 1.28	3.88 ± 0.67	3.73 ± 0.69	5.45 ± 1.64	6.72* ± 1.59	4.47 ± 0.77
Liver/body weight (mg g^−1^)	42.59 ± 2.92	42.20 ± 4.57	39.62 ± 2.99	42.97 ± 1.97	43.67 ± 3.97	43.47 ± 1.61	42.37 ± 5.91	43.80 ± 2.96	36.77 ± 3.20	43.83 ± 3.51
Spleen/body weight (mg g^−1^)	3.61 ± 0.36	3.55 ± 0.27	3.46 ± 0.27	3.60 ± 0.45	4.09 ± 1.02	3.48 ± 0.44	3.44 ± 0.41	3.61 ± 0.52	3.05 ± 0.34	3.50 ± 0.39
Kidney/body weight (mg g^−1^)	6.03 ± 0.47	6.07 ± 0.39	5.68 ± 0.64	5.99 ± 0.32	6.15 ± 0.88	6.26 ± 0.53	6.26 ± 0.42	6.07 ± 0.39	5.61 ± 0.73	5.52 ± 1.96
Colon/body weight (mg g^−1^)	8.08 ± 1.05	8.25 ± 0.84	7.77 ± 0.86	8.36 ± 0.67	9.13 ± 1.09	8.38 ± 0.58	8.68 ± 1.01	8.50 ± 0.46	7.55 ± 0.41	8.84 ± 0.83
Small int./body weight (mg g^−1^)	36.80 ± 1.84	36.39 ± 3.50	36.83 ± 5.11	38.14 ± 3.78	35.88 ± 3.88	36.89 ± 3.59	37.05 ± 4.18	36.19 ± 4.20	31.29 ± 2.33	36.65 ± 3.92

Data represent mean ± SD of C57BL/6J and 5xFAD mice body and organ weights after 3 weeks (A) or 14 weeks (B) exposure to CeO_2_ or SiO_2_ [0.1% or 1%] in feed pellets ad libitum versus the body and organ weights of the corresponding mice that received control feed pellets. Statistical analysis was performed using ANOVA with Dunnett evaluation;

*Versus control feed pellets exposed C57BL/6J mice, *p* < 0.01

^a^Body weight gain at time interval between exposure start and sacrifice

In the C57BL/6J mice that were exposed for 14 weeks to 1% CeO_2_ body weight gain and body weights at sacrifice were found to be significantly augmented in comparison to the corresponding control group (Table 1B). This effect was observed from exposure week 8 onwards (Fig. 1C).

We found no differences in the weights of liver, spleen, kidney, small intestine or colon of the 5xFAD and C57BL/6J mice after 3 weeks or 14 weeks exposure (Table 1). Treatment related effects on length and weight/length ratios of small intestine and colon were also not seen, with one exception: A reduced colon length was observed in the 5xFAD mice after 3 weeks exposure to 1% SiO_2_ (Table 2). However, colon weight and colon weight/length ratio were not significantly different in this group.

**Table 2:** Lengths and weight/length ratios of colon and small intestine

	Control		SiO_2_ [0.1%]		SiO_2_ [1%]		CeO_2_ [0.1%]		CeO_2_ [1%]	
	C57BL/6J	5xFAD	C57BL/6J	5xFAD	C57BL/6J	5xFAD	C57BL/6J	5xFAD	C57BL/6J	5xFAD
*A*										
Colon length (cm)	7.88 ± 0.66	7.77 ± 0.60	7.23 ± 0.65	7.41 ± 0.68	7.81 ± 0.53	6.97* ± 0.54	7.58 ± 0.56	8.00 ± 0.54	6.97 ± 0.89	7.56 ± 0.68
C. weight/length ratio (g cm^−1^)	0.022 ± 0.002	0.021 ± 0.003	0.022 ± 0.002	0.021 ± 0.004	0.020 ± 0.003	0.023 ± 0.003	0.022 ± 0.003	0.022 ± 0.003	0.022 ± 0.003	0.022 ± 0.003
Small intestine length (cm)	31.30 ± 2.47	34.20 ± 2.55	31.48 ± 1.68	33.00 ± 2.66	32.34 ± 2.13	32.11 ± 2.52	31.95 ± 3.05	32.66 ± 2.83	31.55 ± 1.99	31.64 ± 2.43
S.I. weight/length ratio (g cm^−1^)	0.028 ± 0.005	0.024 ± 0.005	0.025 ± 0.004	0.026 ± 0.003	0.024 ± 0.004	0.024 ± 0.008	0.024 ± 0.003	0.026 ± 0.003	0.028 ± 0.002	0.028 ± 0.003
*B*										
Colon length (cm)	8.60 ± 0.85	8.16 ± 0.73	8.32 ± 0.65	8.36 ± 0.55	8.55 ± 0.27	8.16 ± 0.62	8.55 ± 0.43	8.19 ± 0.85	7.80 ± 0.76	8.00 ± 0.51
C. weight/length ratio (g cm^−1^)	0.021 ± 0.004	0.023 ± 0.003	0.022 ± 0.003	0.022 ± 0.002	0.024 ± 0.002	0.022 ± 0.002	0.023 ± 0.003	0.024 ± 0.004	0.025 ± 0.003	0.024 ± 0.003
Small intestine length (cm)	31.88 ± 2.65	31.19 ± 3.08	34.77 ± 1.53	31.79 ± 3.51	31.50 ± 3.12	32.27 ± 1.61	32.47 ± 1.83	33.55 ± 1.36	30.68 ± 1.64	32.22 ± 1.39
S.I. weight/length ratio (g cm^−1^)	0.025 ± 0.003	0.027 ± 0.004	0.025 ± 0.001	0.027 ± 0.003	0.025 ± 0.003	0.025 ± 0.002	0.026 ± 0.003	0.025 ± 0.004	0.026 ± 0.003	0.025 ± 0.003

Data represent mean ± standard deviation of C57BL/6J and 5xFAD mice colon and small intestine length after 3 weeks (A) or 14 weeks (B) exposure to CeO_2_ or SiO_2_ [0.1% or 1%] in feed pellets ad libitum versus the body and organ weights of the corresponding mice that received control feed pellets. C = Colon; S.I. = Small intestine. Statistical analysis was performed using ANOVA followed by Dunnett evaluation

*p < 0.05 versus 5xFAD mice exposed control feed pellets

### Histopathology

Histopathology was performed on liver and spleen, small and large intestine of C57BL/6J mice that were exposed for 14 weeks to evaluate potential treatment-related dose-dependent effects (Table 3). In the liver, increased glycogen was observed in 3 out of 6 mice exposed to 0.1% CeO_2_ and periportal vacuolation in 3 out of 6 mice exposed to 1% CeO_2_. In small and large intestine focal, minimal inflammatory infiltrates were seen, occasionally together with some focal irregular epithelial surfaces. These findings and all other findings seen in the organs evaluated are not assessed to be treatment-related adverse effects.

**Table 3:** Liver and spleen, small and large intestine histopathology

	Control	0.1% SiO_2_	1% SiO_2_	0.1% CeO_2_	1% CeO_2_
Liver	n = 5	n = 6	n = 6	n = 6	n = 6
Focal inflammatory infiltrates	1 (5)	1 (4), 2(2)	1 (6)	1 (6)	1 (6)
Focal necrosis	1 (2)	1 (1)	1 (2)	0 (6)	0 (6)
Increased interstitial cells	0 (5)	2 (1)	1 (1)	1 (1)	1 (1)
Increased glycogen	0 (5)	0 (6)	0 (6)	2 (2), 3 (1)	0 (6)
Vacuolation	0 (5)	0 (6)	0 (6)	0 (6)	2 (3)
Spleen	n = 5	n = 6	n = 6	n = 5*	n = 6
Increased pigment	2 (1)	2 (3)	0 (6)	2 (2)	1 (1), 2 (1)
Congestion	0 (5)	2 (1)	0 (6)	0 (5)	0 (6)
Increased extramedullary hematopoiesis	0 (5)	0 (6)	3 (1)	1 (1)	2 (1)
Increased megakaryocytes	0 (5)	0 (6)	2 (1)	0 (5)	0 (6)
	*One sample not evaluable due to embedding artefacts				
Small and large intestine					
	Minimal focal inflammatory infiltrates (intra-mucosal) in all specimens				
	In some cases, inflammatory infiltrates in adjacent tissue and pancreas with focal vacuolation (grade 2)				
	Clearly visible goblet cells in PAS stained slides (small intestine + ; large intestine +++)				
	Partly mucus on surface. Gut associated lymphoid tissue (GALT) in almost all specimens detectable				

Histopathology of C57BL/6J mice that were exposed for 14 weeks to CeO_2_ or SiO_2_ [0.1% or 1%] in feed pellets ad libitum*.* Shown is the grading of the lesion and the number of animals in brackets. The following grading has been used: 0 = no findings, 1 = minimal, 2 = slight, 3 = moderate, 4 = severe, 5 = massive

### Behaviour

**Fig. 2:**
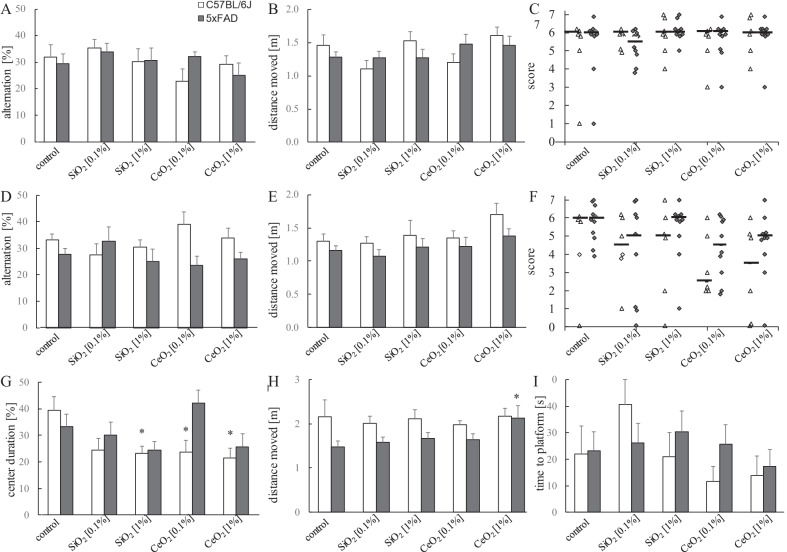
Behaviour tasks performances of C57BL/6J and 5xFAD mice. Mice were exposed for 3 weeks (**A**–**C**) or 14 weeks (**D**–**I**) to SiO_2_ [0.1%, 1%] or CeO_2_ [0.1%, 1%] nanomaterials in food pellets ad libitum. Data represent mean ± SEM of the% alternation and distance moved (m) in the X-maze task (**A**, **B**, **D**, **E**), the % centre duration and distance (m) moved in the open field task (**G**, **H**) and the time to reach the platform (s) of the balance beam test (**I**) for C57BL/6J mice (open squares) and 5xFAD mice (solid squares). String suspension task score data are indicated as scatterplots and median values are indicated as horizontal bars (**C**, **F**) for C57BL/6J mice (open triangles) and 5xFAD mice (solid diamonds). Statistical analysis was performed using ANOVA followed by Dunnett post-hoc evaluation for X-maze, string and balance beam tests. Results of the string suspension task were evaluated by the Kruskal–Wallis test followed by Dunn-Bonferroni post hoc evaluation; *p < 0.01 versus corresponding C57BL/6J or 5xFAD mice exposed to control feed pellets. Number of animals per group: 3 weeks C67BL/6J control (n = 6); SiO_2_ 0.1% (n = 6); SiO_2_ 1% (n = 7); CeO_2_ 0.1% (n = 6). CeO_2_ 1% (n = 6); 5xFAD control (n = 10); SiO_2_ 0.1% (n = 10); SiO_2_ 1% (n = 9); CeO_2_ 0.1% (n = 10). CeO_2_ 1% (n = 10); 14 weeks C67BL/6J control (n = 5); SiO_2_ 0.1% (n = 6); SiO_2_ 1% (n = 6); CeO_2_ 0.1% (n = 6). CeO_2_ 1% (n = 6); 5xFAD control (n = 11); SiO_2_ 0.1% (n = 9); SiO_2_ 1% (n = 10); CeO_2_ 0.1% (n = 10). CeO_2_ 1% (n = 10)

Early memory deficits, followed by successive reduction of other cognitive functions are major characteristics of AD. A battery of behavioural tests was performed to assess for functional neurotoxic effects resulting from the oral exposures to the SiO_2_ and CeO_2_ NMs in the 5xFAD and C57BL/6J littermate mice, as well as to correlate their outcomes with Aβ neuropathology. Results of the behaviour studies are show in Fig. 2.

The X-maze test was used to assess for decreased spontaneous alternation behaviour as an indicator of impaired spatial working memory [33]. Spontaneous alternation is based on the natural behaviour of rodents to explore new environments and thus to rotate in the entries of the arms of the maze. We observed no significant treatment-related effects on spatial working memory in the 3-week and 14-week sub-studies for SiO_2_ and CeO_2_ (Fig. 2A, D). Total distance moved in the X-maze also did not differ between the treatment groups (Fig. 2B, E). The string suspension task was performed to evaluate the agility and grip strength of the mice [34] using a score rating system as described in detail in the methods section. For this test, also no significant differences were identified associated with the exposures to SiO_2_ or CeO_2_ although a trend toward impaired performance was observed in the CeO_2_ exposed C57BL/6J mice in the 14-week sub-study (Fig. 2F).

In addition to the aforementioned tests, in the 14-week sub-study the open field test [35] and the balance beam test were included. In the open field test a decreased proportion of time spent in the central versus the border regions of the arena has been proposed an indicator of increased anxiety. In this study, the WT mice that were exposed for the 14 weeks to 1% SiO_2_ as well as those that were exposed to 0.1% and 1% CeO_2_ spent significantly less time in the central region of the open field arena compared to the control mice (Fig. 2G). In the 5xFAD mice, these treatment-related differences in centre residency times were not observed. However, the 5xFAD mice exposed to 1% CeO_2_ were found to be significantly more active and travelled a greater distance compared to the 5xFAD control mice (1.48 ± 0.45 m for control vs 2.13 ± 0.87 m, *p* = 0.030) indicative of increased locomotor activity (Fig. 2H). In the balance beam test, which was included as an independent indicator of motor coordination and balance [33, 36] the 14-week oral exposures to SiO_2_ and CeO_2_ revealed no significant differences, neither in the 5xFAD mice nor in the WT mice (Fig. 2I).

### Plaque formation

**Fig. 3:**
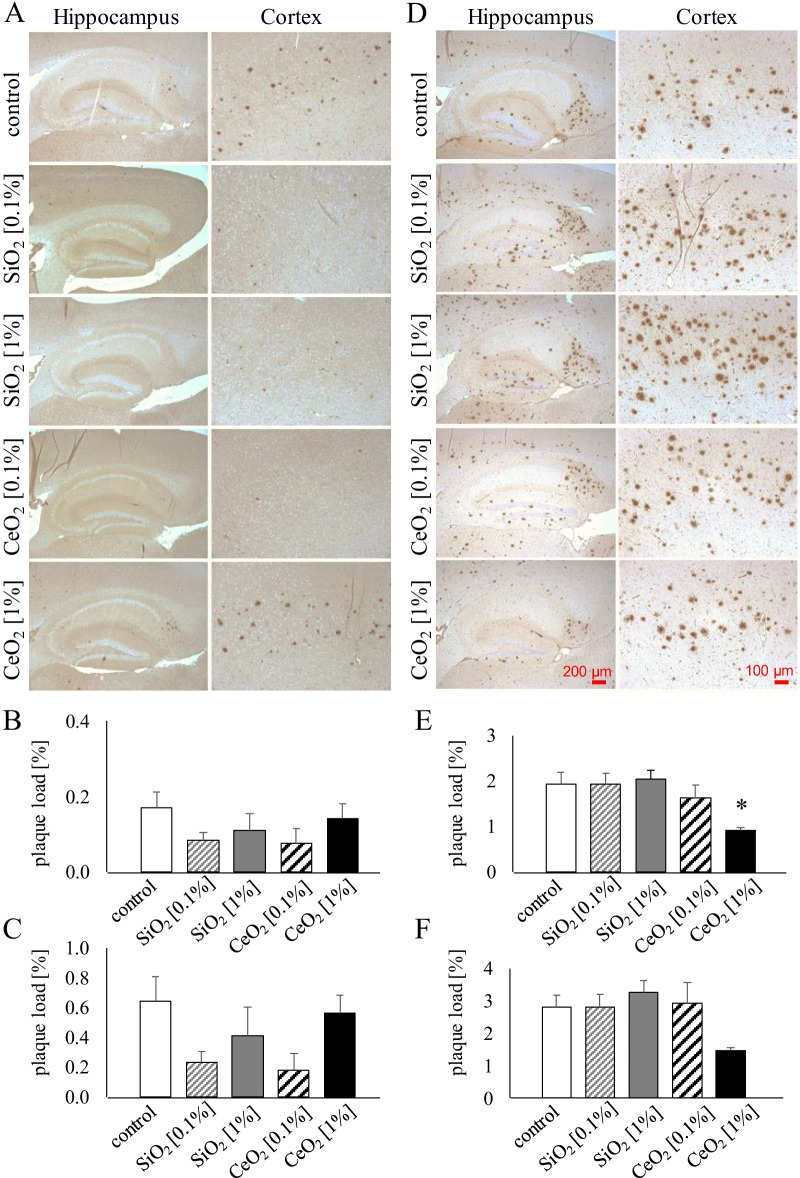
β-Amyloid pathology in 5xFAD transgenic mice. Accumulation of Aβ42 (brown staining) in cortex and hippocampus of 5xFAD mice exposed orally to SiO_2_ [0.1%, 1%] and CeO_2_ [0.1%, 1%] nanomaterials. Representative images of hippocampus and cortex are shown for each treatment after 3 weeks exposure (**A**) and after 14 weeks exposure (**D**). The graphs represent mean ± SEM of plaque load, determined using image analysis software and calculated as the percentage area occupied by Aβ immunostaining in hippocampus (**B**, **E**) and cortex (**C**, **F**) of mice after 3 weeks (**B**, **C**) and after 14 weeks exposure (**E**, **F**). Statistical analysis was performed using ANOVA with Dunnett post-hoc analysis; *p < 0.01 versus mice exposed to control feed pellets. Number of animals per group: 3 weeks 5xFAD control (n = 10); SiO_2_ 0.1% (n = 10); SiO_2_ 1% (n = 9); CeO_2_ 0.1% (n = 10). CeO_2_ 1% (n = 10); 14 weeks 5xFAD control (n = 11); SiO_2_ 0.1% (n = 9); SiO_2_ 1% (n = 10); CeO_2_ 0.1% (n = 10). CeO_2_ 1% (n = 10)

Amyloid β-containing senile plaques are present before clinical symptoms of AD appear [37]. Therefore, parasagittal brain slices of 5xFAD mice were stained with an antibody against human Aβ42 to investigate the impact of the oral exposure to CeO_2_ or SiO_2_ on Aβ plaque load in hippocampus and cortex of the 5xFAD mice. Results are shown in Fig. 3.

As observed in representative images, at younger age (i.e. 3-week exposure study) the 5xFAD mice display much less and smaller plaque formation (Fig. 3A) compared to the older animals (i.e. 14-week exposure study) (Fig. 3D). The relative extent of plaque formation detected in the control animals at the respective ages aligned well with the described accelerating phenotype of the 5xFAD model [32] and findings in previous studies in our lab [9, 38]. In the mice that were exposed for 3 weeks to the lower concentrations (i.e. 0.1%) of SiO_2_ and CeO_2_ tended to show some lower plaque levels, in cortex as well as hippocampus, in comparison to the control mice. However, these differences were not statistically significant. More importantly, the 14-week sub-study, plaque load tended to be decreased in dose-dependent fashion in the hippocampus of the CeO_2_ exposed mice. In the hippocampus as well as in the cortex, plaque load in the 1% CeO_2_ group was approximately half as abundant as in the control group, and statistically significant for hippocampus (ANOVA-Dunnett, *p* < 0.01) but not cortex (*p* = 0.075).

### Amyloid β levels

**Fig. 4:**
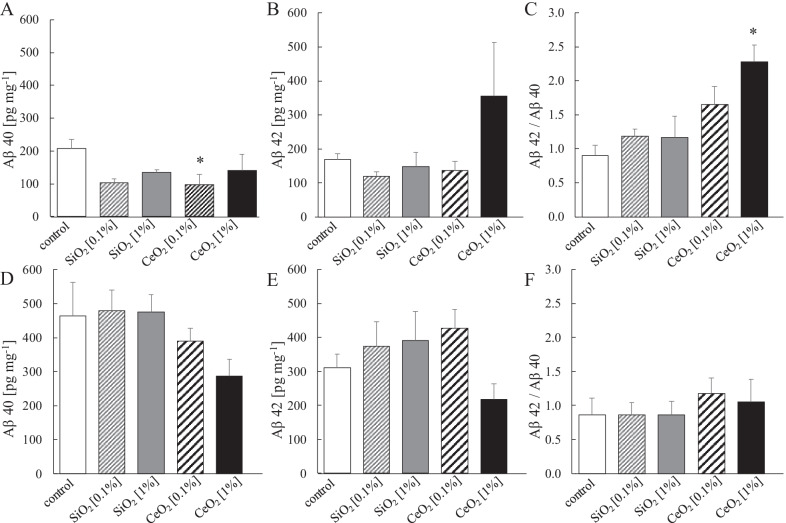
β-amyloid protein levels in 5xFAD transgenic mice exposed to different nanomaterials. Aβ40 (**A**, **D**) and Aβ42 (**B**, **E**) protein levels and Aβ42/Aβ40 ratio (**C**, **F**) were determined by ELISA in cortex brain homogenates of 5xFAD mice after oral exposure to SiO_2_ and CeO_2_ nanomaterials [0.1% and 1%] for 3 weeks (**A**–**C**) or 14 weeks (**D**–**F**). Statistical analysis was performed using ANOVA with Dunnett post-hoc analysis; *p < 0.01 versus mice exposed to control feed pellets. N = 6 animals per group for 3 weeks study and N = 7 for 14 weeks study

To further evaluate effects of the oral exposures to the SiO_2_ and CeO_2_ NMs, cortex lysates were analysed for protein levels of Aβ40 and Aβ42 by ELISA (Fig. 4). In the tissues of the mice that were exposed for 3 weeks to the lower concentration of SiO_2_ and CeO_2_ NMs, protein levels of both Aβ40 and Aβ42 tended to be lowest, in alignment with the histopathological findings (see Fig. 3). The levels of Aβ40 in the 0.1% CeO_2_ group were significantly lower than the controls. Furthermore, the 1% CeO_2_ exposed mice revealed a significantly increased Aβ42/Aβ40 ratio, which was mainly the result of the increased trend of Aβ42 levels in this group. In the 14-week sub-study, levels of Aβ40 as well as Aβ42 were lowest in the cortex tissues of the 1% CeO_2_ group. Although these differences were not significant, they aligned well with the Aβ plaque load findings (Fig. 3). Differences in Aβ42/Aβ40 ratios were not observed at this time point.

### Oxidative stress

**Fig. 5:**
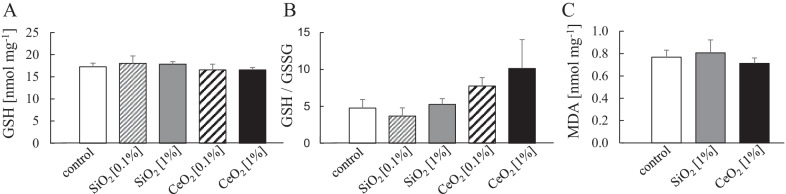
GSH concentrations, GSH/GSSG ratios and MDA concentration in 5xFAD transgenic mice. GSH concentrations (**A**), ratio of GSH/GSSG (**B**) (mean ± SEM, N = 6) in cerebellum after 14 weeks oral administration of SiO_2_ [0.1%, 1%] and CeO_2_ [0.1%, 1%] nanomaterials and MDA concentration (**C**) in midbrain (mean ± SEM, N = 5) after 14 weeks oral administration of SiO_2_ [1%] and CeO_2_ [1%] nanomaterials encapsulated in feed pellets

Oxidative stress resulting from a disruption of pro- and antioxidant balance has been proposed as a major mechanism of neurotoxicity of NMs [2, 39] has also been connected to β-amyloidogenesis and AD pathology [40–43]. To evaluate oxidative stress in the brains of the 5xFAD mice, we measured the levels of glutathione (GSH) [44]. In addition, we determined the ratio of reduced to oxidized glutathione (GSH/GSSG), as reduced ratios have been observed in AD [45, 46]. Results are shown in Fig. 5. The brain tissue levels of GSH were not affected following the subchronic oral exposures to SiO_2_ or CeO_2_. Also, no decreases in GSH/GSSG ratio were observed that would suggest increased oxidative stress in the brain by the nanomaterials. Interestingly, in the brains of the CeO_2_ exposed animals, rather a trend for a dose-dependent increase in GSH/GSSG was noted. However, this effect was not statistically significant. As an independent indicator of oxidative stress, we analysed the levels of the lipid peroxidation marker malondialdehyde (MDA) in selected brain tissue samples (Fig. 5). In alignment with the GSH findings, these results confirmed that neither SiO_2_ nor CeO_2_ cause sustained oxidative stress in the mouse brains.

### Neuroinflammation

**Fig. 6:**
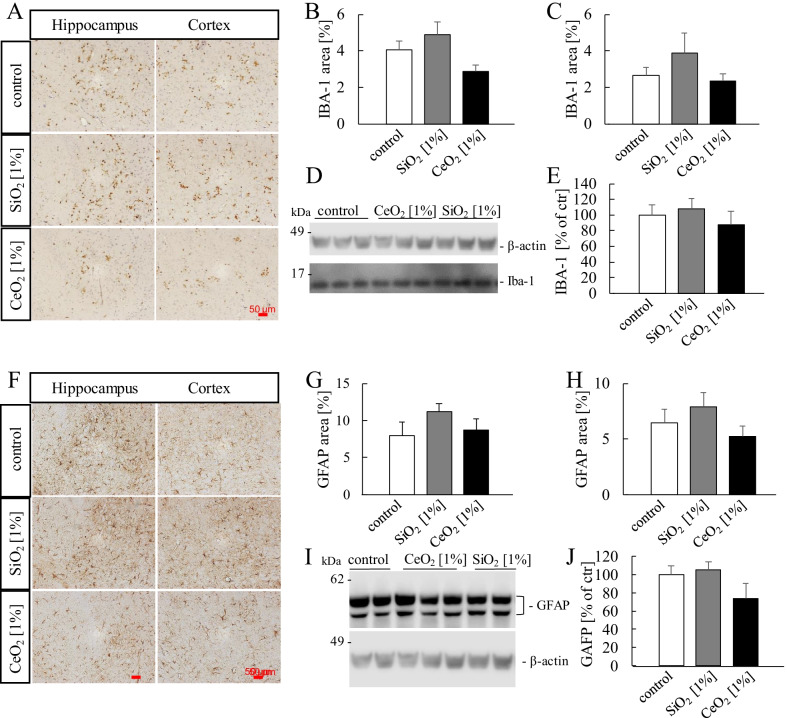
Ionized calcium-binding adapter molecule 1 (IBA-1) and glial fibrillary acidic protein (GFAP) pathology. Representative pictures of IBA-1 (**A**) and GFAP (**F**) (brown staining) in cortex and hippocampus of 5xFAD mice exposed orally to SiO_2_ [1%] and CeO_2_ [1%] nanomaterials. Analyses were performed using image analysis software (ZEN2011, Zeiss) at 200 × magnification. The output of the analyses represents the percentage of positive staining relative to the total area of the cortex or hippocampus and is defined as IBA-1 load for positive stained microglia and GFAP load for positive stained astrocytes in hippocampus (**B**, **G**) and cortex (**C**, **H**). Number of animals per group: GFAP staining control (n = 10); SiO_2_ 1% (n = 8); CeO_2_ 1% (n = 8); IBA-1 staining: control (n = 10); SiO_2_ 1% (n = 3); CeO_2_ 1% (n = 10). IBA-1 and GFAP were also visualized by western blot using FluorChem Imager (**D**, **I**). Total IBA-1 or GFAP was detected in cortex and normalized to the respective control sample (**E**, **J**). Animal numbers: control (n = 10); SiO_2_ 1% (n = 9); CeO_2_ 1% (n = 10)

Neuroinflammation is a crucial pathological hallmark and mediator of neurodegenerative diseases including AD. We therefore evaluated the expression of ionized calcium-binding adapter molecule 1 (IBA-1) and glial fibrillary acidic protein (GFAP) in the brains of the 5xFAD mice after the subchronic exposures to SiO_2_ and CeO_2_. Increased IBA-1 expression is an indicator of activated microglia in the brain under conditions of inflammation [47] and therefore used as marker of neuroinflammation﻿. The expression of GFAP is upregulated in most forms of reactive astrogliosis [48]. The results of the IBA-1 and GFAP analyses are shown in Fig. 6. As shown by representative immunohistochemical staining images from sections of paraffin-embedded brain hemispheres (Fig. 6A), no significant effects of the oral exposures to the NMs on IBA-1 expression were found in hippocampus (Fig. 6B) or cortex (Fig. 6C). For the cortex region this was also confirmed using Western blot analysis (Fig. 6D, E). Similarly, neither SiO_2_ nor CeO_2_ caused increased expression of GFAP. The expression of this astrocyte marker did not differ between the exposure groups as revealed by immunohistochemical analysis in hippocampus (Fig. 6F, G) and cortex (Fig. 6F, H) and independently by Western blot detection (Fig. 6I, J). Taken together, in alignment with the findings on Aβ plaque formation, Aβ peptide levels and oxidative stress markers, neither SiO_2_ nor CeO_2_ caused neuroinflammation upon long-term oral exposure.

## Discussion

The present work was undertaken to address if long-term oral exposure to two of the most commonly used NMs, SiO_2_ and CeO_2_ can cause neurotoxicity and promote AD. The findings suggest that long-term oral exposure to these NMs has no adverse health impact on the central nervous system but, by contrast, support a potential anti-amyloidogenic role of CeO_2_ in Alzheimer’s disease.

With regard to SiO_2_, our findings are of main relevance in view of its use as a food additive. The amount of use of SiO_2_ in food applications is limited to 2% by the US Food and Drug Administration [31], while the European Food Safety Authority (EFSA), depending on the food category, authorizes the use of E551 *quantum satis* or mostly at a maximum permitted level (MPL) of 1%, with the exception of foods for infants and young children [19]. While EFSA concluded that there is no indication of a risk when used as a food additive, in a recent study in mice adverse effects were observed following 18 months exposure via drinking water [49]. In the present study, mice were exposed to SiO_2_ incorporated in the food. This differs from exposure via drinking water and the most commonly used administration by repeated gavage [12, 50]. Based on an estimated daily feed consumption of 4 g and average mouse body weight of 20 g, the ad libitum exposure to the dosed feed pellets (0.1 and 1% w/w) result in a daily intake of about 0.2 and 2 g kg^−1^ bodyweight (BW). Up to the highest 14-week cumulative dose, the dietary exposure to SiO_2_ NMs did not cause accelerated plaque formation, oxidative stress, neuroinflammation, spatial working memory deficits, locomotor activity changes or motor coordination impairments. Solely, for the wildtype C57BL/6J mice an effect in the open field test was observed for the 14-week 1% SiO_2_ group. As this effect was not seen in 5xFAD mice and not accompanied with any further effects, it can be debated whether this reflects an adverse neurotoxic response. When applied in an unconditioned manner, the open field test has been suggested to reflect other effects than anxiety, like avoidance or natural preference responses [51].

The findings from our in vivo study in the 5xFAD model are in contrast to in vitro papers that suggest potential amyloidogenic effects of SiO_2_ NMs [52, 53]. However, when investigating such direct effects of NMs on cells in vitro*,* it should be kept in mind that in vivo research has shown that the translocation and accumulation of SiO_2_ into the brain following oral exposure is extremely low, if at all [54, 55]. Peripheral effects, including those in intestine, liver and spleen as major recognized target organs for ingested NMs, were also found to be mostly absent. However, a significantly diminished body weight gain was observed, exclusively, in the 5xFAD mice of the 1% SiO_2_ group. This effect was first apparent at the 5th week of exposure but no longer present after week 14 as sacrifice. In association with this, a lower colon length was found in the 1% SiO_2_ exposed 5xFAD mice after 3 weeks exposure, but not after 14 weeks. This may point towards a (transient) increase in susceptibility to local intestinal effects of SiO_2_ NMs in the 5xFAD model compared to the C57BL/6J mice. Indeed, in support of this hypothesis, differences in intestinal gene expression, trypsin levels, faecal microbiota composition and associated weight changes have been shown between 5xFAD mice and their wildtype littermates [56, 57].

Similar to SiO_2_, the oral exposure to the other NM that we chose to investigate, CeO_2_, did not result in adverse neurotoxic and AD-promoting outcomes. On the contrary, a marked anti-amyloidogenic effect was found in the 5xFAD mouse model. Unlike SiO_2_, CeO_2_ presently finds no intentional application in the food sector. However, its potential use in disease prevention, therapy and diagnostics has been promoted in several recent oral exposure studies in rodents, for instance, in models of non-alcoholic fatty liver disease [58] and colitis [59, 60].

To the best of our knowledge, this study is the first to demonstrate an inhibition of AD-like pathology following long-term oral exposure to CeO_2_ NMs. Specifically, in the 14-weeks exposed mice, the Aβ plaque load was approximately 50% lower in both hippocampus and cortex of the 1% CeO_2_ fed 5xFAD mice compared to the corresponding control group. In alignment with these pronounced immunohistopathology findings, cortical protein levels of Aβ40 and Aβ42 tended to be markedly lower in the 1% CeO_2_ group as well, albeit not statistically significant. Since our study was a priori designed to address the potential adverse effects of long-term oral exposures to NMs, we can only speculate about underlying mechanisms of the observed beneficial effects of the CeO_2_ feeding. While amyloid pathology in AD has been linked to oxidative stress and inflammation [61–63], the reduced plaque burden in the 1% CeO_2_ exposed mice was not accompanied by significant changes in oxidative stress or neuroinflammation. Yet, it was interesting to observe the highest GSH/GSSG ratio in the brains of the 1% CeO_2_ group. Lower GSH/GSSG ratios have been observed in AD [45, 46] and an increase might thus reflect a compensatory improved antioxidant status in the CeO_2_ fed 5xFAD mice. Notably, the lower amyloid plaque burden was apparent after the 14-week cumulative exposure to CeO_2_ despite the aggressive phenotype of the 5xFAD model. Using the same mouse model, we previously demonstrated a rapid acceleration of plaque formation following a 3-week inhalation exposure to diesel exhaust, representing a dominant contributor of nano-size air pollution particles in urban environments [9]. At 13-weeks exposure, the plaque promoting effect of the diesel exhaust was no longer present, most likely due to the strong age-dependent progressive nature of the 5xFAD model [9]. In our present study, a beneficial effect of the CeO_2_ was not yet observed after 3 weeks, which could be due to the low absolute plaque load in cortex and hippocampus at this young age. Interestingly, however, at this time point a significant increase in Aβ42/Aβ40 ratio was detected in the 1% CeO_2_ exposed 5xFAD mice, mainly as a result of the relatively higher levels of the more toxic and aggregation prone Aβ42 protein [64, 65]. Whether and how this seemingly contrasting finding at early exposure could relate to the lower formation of plaques at the later 14-week exposure needs further research. In another recent study, we investigated the neurotoxic and AD-promoting effects of CeO_2_ NMs doped with varying amount of zirconium (ZrO_2_) in a 4-week inhalation design in 5xFAD mice [38]. Here, unlike diesel exhaust, these CeO_2_ containing NMs did not lead to an aggravated plaque formation following inhalation exposure and, unlike our current oral exposure study, also did not inhibit plaque formation in 5xFAD mice. While this may be explained by differences in levels and duration of exposure, it also demonstrates the likely importance of the route of exposure.

As a redox-sensitive nanomaterial, CeO_2_ has been long recognized for its free radical scavenging properties and, therefore, is widely studied for its potential as an antioxidant and anti-inflammatory agent in the field of nanomedicine [66–68]. Several research groups have already investigated the neuroprotective properties of CeO_2_ NMs and explored their potential therapeutic use in brain diseases. In a rat model of Parkinson’s disease, intrastriatal injection of CeO_2_ NMs could attenuate neurobehavioral impairments [69]. In a mouse model of multiple sclerosis, intravenous administration of citrate/EDTA-stabilized CeO_2_ ameliorated motor function deficits [70]. Interestingly, Kwon and co-workers [71] revealed therapeutic promise for triphenylphosphonium-conjugated CeO_2_ in AD by showing a suppression of reactive gliosis and mitochondria damage in 5xFAD mice upon stereotactic injection. However, in contrast to our findings, they did not observe a significant attenuation of plaque load in these mice.

While increasingly complex nanomedicine-based strategies are being proposed and developed for AD [72, 73], it was striking to observe the effects in our study (1) with pristine, non-stabilized/conjugated CeO_2_, and (2) by a mere dietary exposure instead of a forced intravenous or intracranial administration. It is tempting to conclude that the effects observed with the CeO_2_-fed mice resulted from direct redox-restoring effects of these NMs, as suggested from in vitro investigations. Indeed, CeO_2_ NMs were shown to reduce ROS generation in neuronal cell cultures and to block mitochondrial fragmentation produced by Aβ [71]. Hybrid nanoparticles composed of ceria and polyoxometalate were shown to degrade Aβ aggregates and reduce intracellular ROS in PC12 cells [74]. As our study did not include a pharmacokinetic design, it is not known to what extent the CeO_2_ NMs may have reached and accumulated in the brain of the mice. Major progress in this specific research area has been achieved previously by Yokel and colleagues. Using CeO_2_ NMs of different primary size, they demonstrated that liver and spleen are major target organs in rat after a single intravenous administration, while only a small proportion of the dose enters the brain [75]. More recently, they demonstrated that translocation of CeO_2_ NMs from the lung to the rest of the body is less than 1% of the deposited dose and that translocation from the gastrointestinal is even lower [76]. However, they also observed that the organ burdens of the translocated fractions persisted for at least months, suggesting very slow clearance rates. Several other groups have confirmed the minimal to absent absorption of CeO_2_ NMs from the gastrointestinal tract of rats [77] and mice [78, 79]. Therefore, it should also be considered that the anti-amyloidogenic effect observed in our present study may be the result of peripheral effects of CeO_2_. Future studies on the neuroprotective effects of nanoceria should therefore also aim at the investigation of their effects on organs and tissues other than the brain.

In our study, indications of peripheral adverse effects of the CeO_2_ NMs were merely detected in the C57BL/6J mice. As a main finding, in these wildtype animals, a significant increase on body weight gain was observed during the 14 weeks with the 1% dosed pellets. Subsequent histopathology analysis revealed increased glycogen in 3 out of 6 animals of the 0.1% CeO_2_ group and periportal vacuolation in 3 out of 6 animals of the 1% CeO_2_ group. Changes in the weights of liver, spleen and kidney, as well as weights, lengths and weight/length ratios of colon and small intestine were absent. The observed histological findings in the livers of the CeO_2_ fed mice are likely features of increased glycogen storage are therefore considered to be of no toxicologic significance. In contrast to our study, Yokel and co-workers recently found no increased liver vacuolization in C57BL/6 mice after a single intraperitoneal injection of CeO_2_ NMs, and even a decreased vacuolation in BALB/c mice [80]. In the behavioural studies, the only statistically significant effect observed in the C57BL/6J mice with CeO_2_ NMs at 0.1% and 1% was a diminished time spent in the centre of the open field arena. As already mentioned with regard to the comparable observations with SiO_2_, it can be debated whether the findings of this unconditioned test should be interpreted as an anxiety indicator or a mere change in the natural preference response [51].

As opposed to the C57BL/6 J mice, in the 5xFAD mice that were fed with 0.1% or 1% CeO_2_ NMs for up to 14 weeks no significant changes in body weights were found. Histopathology was not evaluated in these transgenic animals, but differences in organ weights, including length and weight/length ratios of small intestine and colon were not observed. This suggests that the beneficial plaque inhibiting effect occurred in the absence of any substantial peripheral toxicity. Moreover, behavioural changes were absent in all tests at both time points of investigation (i.e. week 3 and 14), except for the open field test. Here, the 1% CeO_2_ exposed 5xFAD mice at week 14 were found to be much more active compared to the corresponding 5xFAD controls. Interestingly, the distance covered in the open field test by the CeO_2_ fed 5xFAD animals was highly similar with that of the C57BL/6J controls (i.e. 2.13 ± 0.87 m versus 2.16 ± 0.84 m). Accordingly, it can be suggested that this activity change reflects an improved behaviour as a result of the inhibited plaque load following CeO_2_ exposure. Recently, the 5xFAD model has been proposed as a useful model to study motor dysfunction in AD [81]. Indeed, in line with our investigations, 5xFAD mice travel shorter distances in the open field test than WT mice with increasing age. Taken together, these initial findings in CeO_2_ exposed 5xFAD mice indicate a possible beneficial effect on AD-like pathology. However, before any further indication of a potential therapeutic or preventive use of orally administered CeO_2_ NMs should be given, designated pharmacokinetic and pharmacodynamic studies are needed, preferably using independent (rodent) AD models. This, of course, then also requires an in-depth biocompatibility evaluation.

## Conclusions

Our present study was designed to test the hypothesis that long-term oral exposure to NMs can cause neurotoxicity and aggravate the pathogenesis of AD. It was demonstrated that neither synthetic amorphous SiO_2_ nor CeO_2_ increases amyloid-β plaque formation, neuroinflammation and oxidative stress in 5xFAD Alzheimer model mice in a subchronic dietary exposure design. Behavioural analyses also revealed an absence of spatial working memory deficits and motor coordination impairments. Surprisingly, the subchronic exposure to 1% CeO_2_ containing feed pellets resulted in a marked inhibition of plaque burden in the 5xFAD mice and increased locomotor activity. Summarizing the results, the findings from the present study suggest that long-term oral exposure to synthetic amorphous silica NMs, which find wide applications in the food sector, has no major adverse health impact on the central nervous system, specifically regarding the development or progression of the neurodegenerative Alzheimer’s disease. The observations with CeO_2_ warrant further investigations to explore if long-term dietary administration of this redox-active NM could have beneficial effects in AD.

## Methods

### Nanomaterials

The CeO_2_ JRC reference nanomaterial NM-212 was purchased from the Fraunhofer Institute for Molecular Biology and Applied Ecology (IME, Schmallenberg, Germany). Detailed characteristics of the CeO_2_ NM-212 are provided in the JCR nanomaterial characterisation report [82]. The amorphous fumed SiO_2_ NM sample was obtained from Sigma-Aldrich, Munich, Germany (#S5130). This sample has been previously characterised in detail [83]. To check the particle size for the material batches applied in this study, the primary particle size distributions of the pristine NMs were determined using scanning electron microscopy (SEM) by measuring 425 primary particles (CeO_2_) and 500 particles (SiO_2_), respectively. The CeO_2_ NMs had a mean size of 35.4 nm ± 17 nm following a log-normal size distribution with a mode diameter of 28.7 nm and sigma = 1.38, obtained by a mathematical fit of the size distribution. The particles displayed a nearly spherical particle morphology. The analysis of the SiO revealed a spherical morphology with a mean size of 12.9 ± 4.9 nm again following a log-normal size distribution with a mode diameter of 11.0 nm and sigma = 1.32. Both materials were present in form of bigger agglomerates consisting usually of several ten to hundreds primary particles.

### Study design

The oral exposure studies were performed in heterozygous 5xFAD mice and their nontransgenic C57BL/6J littermates. The 5xFAD mouse model (Jackson Laboratories) carries five familial AD mutations and is characterized by an early onset of AD-related pathology: the double Swedish mutation (K670N/M671L), which is responsible for the enhanced amyloid production, and mutations which are responsible for altered amyloid precursor protein processing leading to a higher ratio of the more amyloidogenic Aβ production such as the Florida (I716V) and London (V717I) mutations in APP and the mutant presenilin 1 (M146L + L286V) with neuronal expression driven by the neuron-specific mouse Thy-1 promoter [32]. Amyloid deposition starts in the deep layers of the cortex and subiculum at 2 months of age, while memory and motor deficits become detectable from 4 to 6 months of age [32, 33]. The mice were handled according to guidelines of the Society for Laboratory Animals Science (GV-SOLAS) and were housed under standard conditions with access to food and water ad libitum. Lighting was artificial with a sequence of 12 h light and 12 h dark. The study was approved by the Landesamt für Natur, Umwelt und Verbraucherschutz (LANUV, NRW, Germany; Ref. no. 84-02.04.2013.A443).

**Fig. 7:**
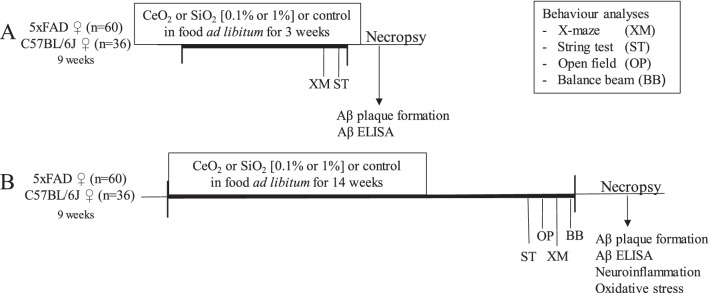
Study design. Nine weeks old female C57BL/6J or 5xFAD mice were fed ad libitum for 3 weeks (**A**) or 14 weeks (**B**) with feed pellets loaded with 0.1% or 1% (w/w) CeO_2_ or SiO_2_ nanomaterials (NMs) or control feed pellets. Motor function, memory and anxiety were determined in a series of behaviour tests performed in the weeks before the necropsies, as follows for the respective substudies: The X-maze test (XM) was performed on exposure day 19 (**A**) or 95 (**B**), the string test (ST) was performed on day 20 (**A**) or 88 (**B**), the open field test (OP) was performed on day 91 (**B**) and the balance beam test (BM) was performed on day 96 (**B**). Aβ plaque formation and Aβ ELISA (**A**, **B**) or markers of neuroinflammation and oxidative stress (**B**) were analysed in the brains of the mice after necropsy

Nine weeks old female 5xFAD and female C57BL/6J littermates were exposed ad libitum for 3 or 14 weeks to feed pellets that were loaded with 0.1% or 1% (w/w) SiO_2_ NM or CeO_2_, or to control feed pellets (Fig. 7). The study was designed with n = 160 mice, i.e. for the respective sub-studies n = 50 5xFAD mice (n = 10 per treatment group) and n = 30 WT mice (n = 6 per treatment group). One 5xFAD mouse (0.1% SiO_2_ exposed) died in the first week of exposure and thus were excluded from all analyses. Moreover, in the 3-week sub-study one WT mouse exposed to 1% SiO_2_ was inappropriately labelled as 5xFAD mouse, whereas in the 14-week study one control 5xFAD mouse was inappropriately classified as WT animal. The study design is shown in Fig. 7. The feed pellets were prepared and provided by ssniff GmbH, Soest, Germany. Additional file 1: Figure S1 shows representative SEM images of the SiO_2_ and CeO_2_ NMs (Fig. S1A,B) within prepared feed pellets and by comparison of the pristine NMs (see also 5.1). Energy dispersive x-ray analysis was used to verify the presence of cerium (Additional file 1: Figure S2) and silicon (Additional file 1: Figure S3). One week prior to study start mice were randomized according to age and body weight. During the weeks before dissection, behavioural studies were performed to assess for effects on anxiety, motor performance and spatial working memory. Following sacrifice, brain tissues were collected as well as further organs for analyses as described below.

### Behavioural tests

In alignment with animal ethics, requirements and routine of our animal facility and previous studies [9, 12, 38] all behaviour tasks were performed during daytime, i.e. during the resting phase of the animals. Motor function and grip strength were tested using the string suspension task, where mice are permitted to grab a string that is suspended between two platforms with their forepaws and subsequently allowed to move to one of these platforms [33, 34]. To rate motor performance during 60 s trials, a scoring system from 0 to 7 was used which was adapted from [84] and described in detail in our previous work [12, 38]. Spatial working memory by spontaneous alternation behaviour was assessed using an open arm cross (X)-maze task as described in Jawhar and colleagues [33] and recent work of our studies [9, 12, 38]. During 5 min test sessions, spontaneous alternation was measured and defined as successful if a mouse visited all of the 4 arms alternately. An impairment in spatial working memory is defined by decrease in spontaneous alternation [85]. Anxiety and exploratory activity was measured using the open field test (Noldus, the Netherlands) [35] as previously described [12]. Increased anxiety was defined by spending less time in the open central area compared to the more hidden border during 5 min test sessions. The balance beam walking assay is used to test motor coordination and balance in rodents as previously described [33, 36]. Therefore, a 50 cm long wooden beam was suspended between two plastic platforms (9 cm × 15 cm) placed above two vertical poles at a height of 40 cm. The mice were released in the middle of the beam and released thereafter allowing the mice to traverse the beam. Performance on the walking assay is quantified by measuring the time it takes for the mouse to escape to one of the platforms during a 60 s trial. The trial was repeated three times in 1 day of testing. If an animal remains on the beam for whole 60 s and does not escape to one of the platforms, the maximum time of 60 s is recorded. All three trials are averaged. To avoid odour distraction, all behaviour tasks were cleaned between trials with 70% ethanol. Behaviour tests were recorded with an infrared camera and analysed with associated software (EthoVision XT 11, Noldus).

### Dissection, tissue preparations and histopathology

The mice were sacrificed by cervical dislocation, followed by decapitation. Right brain hemispheres were stored in 4% paraformaldehyde (PFA) (Merck, St. Louis, Missouri, USA) for immunohistochemistry. Left brain hemispheres were rapidly dissected into cerebellum, midbrain and cortex including hippocampus, snap frozen in liquid nitrogen and then stored at − 80 °C until processing for biochemical analyses. Liver, spleen and kidneys were removed and weighed. Small intestines and colons were removed, flushed with saline and opened, subsequently analysed for weight and length and used to prepare Swiss-rolls [86]. Histology analyses were performed according to routine procedures (fixation in 4% PFA and paraffine embedding). Sections of small and large intestines, liver and spleen were blindly evaluated by an experienced veterinary pathologist using Haematoxylin and Eosin (H&E) stained sections for these organs and additionally Periodic Acid Schiff (PAS) stained sections for small and large intestine. The slides were evaluated semi-quantitatively applying the following grading score: 0 = no findings; 1 = minimal; 2 = slight; 3 = moderate; 4 = severe; 5 = massive.

### Immunostaining of paraffin-embedded brain tissue sections

Immunostaining was performed using antibodies for Aβ 42 (clone G2-11, Cat.N0. MABN12, Merck Millipore, Darmstadt, Germany), IBA-1 (Cat No. GTX100042, GeneTex, Irvine, California, USA) and GFAP (Cat No. Z0334, Dako Agilent, Santa Clara, USA). After sacrificing the mice and careful dissection of the brains, the right hemisphere was processed, as follows. For fixation, the tissue was immediately immersed in 4% buffered PFA at 4 °C for a minimum of 24 h and 3 µm paraffin-embedded sections were subsequently cut on glass. The sections were deparaffinized in xylene (Roth, Karlsruhe, Germany) and rehydrated in a series of ethanol (Roth) baths. To block endogenous peroxidases, sections were pre-treated with 0.3% H_2_O_2_ (Merck, St. Louis, Missouri, USA) in 0.01 M phosphate buffered saline (PBS). Antigen retrieval was generated by boiling slices in 10 mM citrate buffer followed by a 3 min incubation in 88% formic acid (Roth). A solution of 10% FCS (Merck) and 4% milk powder (Roth) in 0.01 M PBS was used to block unspecific antigens. Slices were incubated in primary antibody diluted (1:500 for IBA-1, 1:1000 for Aβ, 1:2000 for GFAP) in 0.01 M PBS and 10% FCS. GFAP and IBA-1 slices were incubated at 4 °C while Aβ-immunostaining was incubated at RT overnight. Next day the sections were washed and incubated 1.5 h at 37 °C for Aβ and 45 min at RT for GFAP and IBA-1 with biotinylated secondary antibody (Vectorlabs, Burlingame, California, USA), diluted 1:200 in 0.01 M PBS and 10% FCS. The counterstaining with Haematoxylin led to a blue staining of the nucleus. Positive antibody staining was visualized using the Avidin–Biotin-Complex-method (ABC) by Vectastain kit (Vectorlabs) and diaminobenzidine (DAB) as chromogen which resulted in a brown colour. Images were taken with a Zeiss Axiophot light microscope equipped with AxioCam MRc (Carl Zeiss, Jena, Germany) and analysed using image analysis software by colour deconvolution algorithm of brown pixels (ZEN2011, Carl Zeiss). The percentage of positive staining relative to the total area represents plaque, GFAP or IBA-1 load and was analysed in cortex and hippocampus.

### Aβ extraction from brain samples and ELISA

Water-soluble Aβ levels were analysed by Enzyme Linked Immunosorbent Assay (ELISA) in cortical cytosolic fractions [87]. To evaluate soluble proteins, brain tissues were homogenized in ~ 8 volumes of ice-cold PBS and supernatants were subsequently frozen at − 80 °C until further analysis. The amount of Aβ 40 and Aβ 42 was determined using an ELISA kit (FUJIFILM Wako Chemicals Europe GmbH, Neuss, Germany) according to the manufactures protocol and normalized to the total protein content in the respective sample [pmol g^−1^ tissue]. Total protein content was determined by PierceTM BCA Protein Assay Kit (Thermo Scientific, Waltham, Massachusetts, USA) as described by the manufacturer.

### Oxidative stress markers

Lipid peroxidation was determined in midbrain tissues by the reaction of MDA with thiobarbituric acid (TBA) to form a colorimetric (532 nm)/fluorometric (λ_ex_ = 532/ λ_em_ = 553 nm) product, proportional to the MDA present. The amount of MDA was evaluated with the MDA kit (Merck) according to the manufactures protocol. The amount of total and oxidized glutathione was evaluated in cerebellum after homogenization in cold 100 mM phosphate buffer (pH 6.8), containing 0.1 mM EDTA (Merck). After centrifugation (10,000*g*, 15 min, 4 °C) the supernatants were deproteinized with an equal volume of 10% metaphosphoric acid (Merck) and thereafter with a solution of 4 M triethanolamine (Merck) to increase the pH of the sample. This assay is based on the catalytic reaction of GSH with 5,5′-dithio-bis (2-nitrobenzoic acid) (DTNB, also named as Ellman’s reagent) that forms the yellow derivate 5-thionitrobenzoic acid (TNB). The concentration of GSH in a sample is proportional to the rate of formation of TNB, measured at 412 nm. The concentration of total glutathione was expressed as nmol tGSH per mg of protein. In addition, oxidized GSH (GSSG) was measured using 2-vinylpyridine for masking GSH which is rapidly reduced in two GSH by glutathione reductase and NADPH. The ratio of reduced glutathione to oxidized glutathione was expressed as (GSH/GSSG). Total protein content was determined by PierceTM BCA Protein Assay Kit (Thermo Scientific, Waltham, Massachusetts, USA) as described by the manufacturer.

### Western blot analysis of IBA-1 and GFAP

For the analysis of protein levels, cortex tissues were homogenized in ~ 8 volumes of ice-cold 0.01 M PBS in a potter tissue grinder. The homogenate was centrifuged in a microcentrifuge for 45 min at 12,500 rpm and 4 °C. The amount of protein in the supernatant was evaluated with the BCA kit (Thermo Scientific) according to the manufactures protocol. The samples were prepared with equal amounts of protein (40 µg) and loaded on a 4–12% precast NUPAGE gel (Invitrogen Thermo Scientific) and were separated at 180 V in a Mini-PROTEAN II tank (BIO-RAD, Hercules, California, USA). After electrophoresis the proteins were blotted on a 0.45 µm pore diameter nitrocellulose transfer membrane (Whatman, Schleicher & Schuell) at 250 mA for 45 min in a Mini Trans-Blot tank (BIO-RAD). The membrane was blocked with 5% milk in PBS-T (0.01 M PBS and 0.05% Tween-20) for 30 min. After the blocking, the membrane was incubated overnight at 4 °C with the primary antibody, i.e. (GFAP (Cat No. ab7260, Abcam, 1:1000) or IBA-1 (Cat No. GTX100042, Gentex, 1:1000). Next day the membrane was washed with PBS-T and was incubated 1 h at room temperature with the horseradish peroxidase-conjugated secondary antibody and washed again 5 times with PBS-T. For the detection of the proteins the electrochemiluminescence (ECL) solutions were applied (GE Healthcare Amersham, Fisher Scientific, Schwerte, Germany), and the visualization was performed with the FluorChem 8900 (Biozym, Hessisch Oldendorf, Germany). Quantification of protein expression was done using the ImageJ software (National Institutes of Health, Bethesda, USA).

### Statistical analyses

All data are shown as mean and standard error of mean (SEM) unless specified otherwise, with the number of animals indicated in the figure legends for each endpoint. Treatment related effects were analysed using one-way analysis of variance (ANOVA) followed by Dunnett’s post hoc evaluation of control groups versus NMs exposed groups. For the evaluation of ordinal data, indicated as scatterplots with median values, the Kruskal–Wallis test with Dunn-Bonferroni post hoc analysis was used. Analysis were performed using SPSS statistics (V25 IBM Corporation, USA).

## Supplementary Information

**Additional file 1**. Figure S1 shows representative SEM images of the SiO_2_ and CeO_2_ NMs (Fig. S1A,B) within prepared feed pellets and by comparison of the pristine NMs (see also 5.1). Figure S2; Figure S3.

## Data Availability

The datasets used and/or analysed during the current study are available from the corresponding author on reasonable request.
