# Supplementary material for: Effects of subchronic dietary exposure to the engineered nanomaterials SiO2 and CeO2 in C57BL/6J and 5xFAD Alzheimer model mice
Source: Part Fibre Toxicol. 2022 Mar 25;19:23. doi: 10.1186/s12989-022-00461-2 (PMC8957165; doi:10.1186/s12989-022-00461-2)
Supplement: Supplementary file 1 — Additional file 1. Figure S1 shows representative SEM images of the SiO2 and CeO2 NMs (Fig. S1A,B) within prepared feed pellets and by comparison of the pristine NMs (see also 5.1). Figure S2; Figure S3. [file 12989_2022_461_MOESM1_ESM.docx]

**Supplement 1**

***
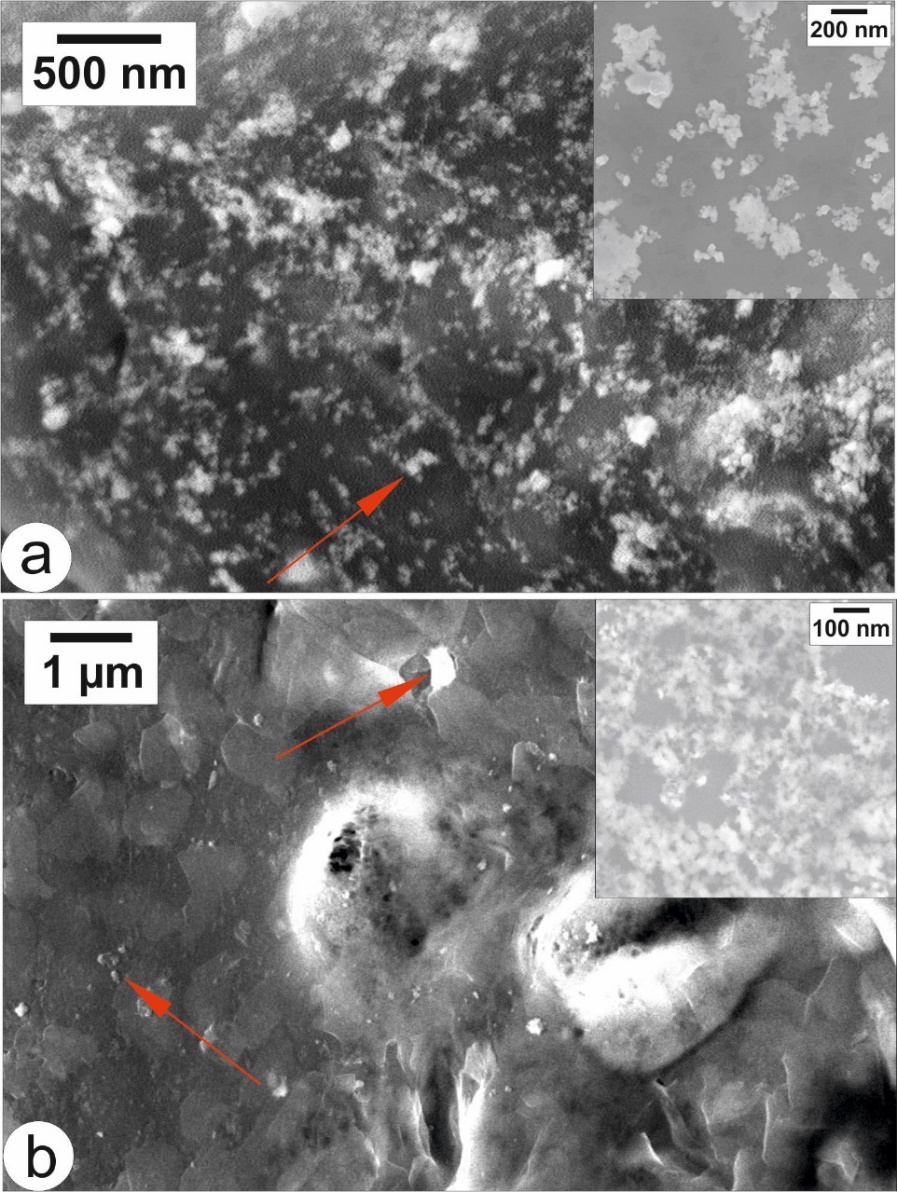
***

**Figure S1.** Scanning electron microscope images of feed pellets loaded with a) CeO_2_ NM and b) SiO_2_ NM. The red arrows point to the particles visible by their brighter contrast. The inserts show the pristine NMs in a higher magnification.

**Method**: To analyse the added NMs and the feed pellets high resolution scanning electron microscope (SEM) images were obtained. For the pristine NMs a JEOL 7500F high resolution SEM (JEOL (Germany) GmbH) was used. The NMs were dispersed in deionized water and applied onto single crystalline silicon wafer pieces. After drying the SEM images were obtained at an acceleration voltage of 5 kV ^[1]^. The SEM investigations of the feed pellets were conducted on the pellets with the 1% NMs using a Tescan CLARA RISE (Tescan GmbH, Dortmund, Germany) high-resolution scanning electron microscope at an acceleration voltage of 15 kV. The feed pellets were not covered with a conductive layer due to the size of the nanoparticles. The images shown in Figure S1 were obtained by using the 4-quadrant backscatter detector revealing the elemental composition of the sample (z-contrast), thus emphasizing the added nanoparticles. The composition was cross-checked by applying energy dispersive x-ray spectroscopy (EDS) analysis (EDAX Octane Elect detector, AMETEK GmbH, Wiesbaden, Germany) verifying the presence of cerium (see Figure S2) and silicon (see Figure S3), respectively. The SEM images show that the added NMs are distributed over the surface of the feed pellet ingredients both at the surface of the pellets as well as inside the pellets. The nanoparticles are usually well dispersed with only a few agglomeration areas present.

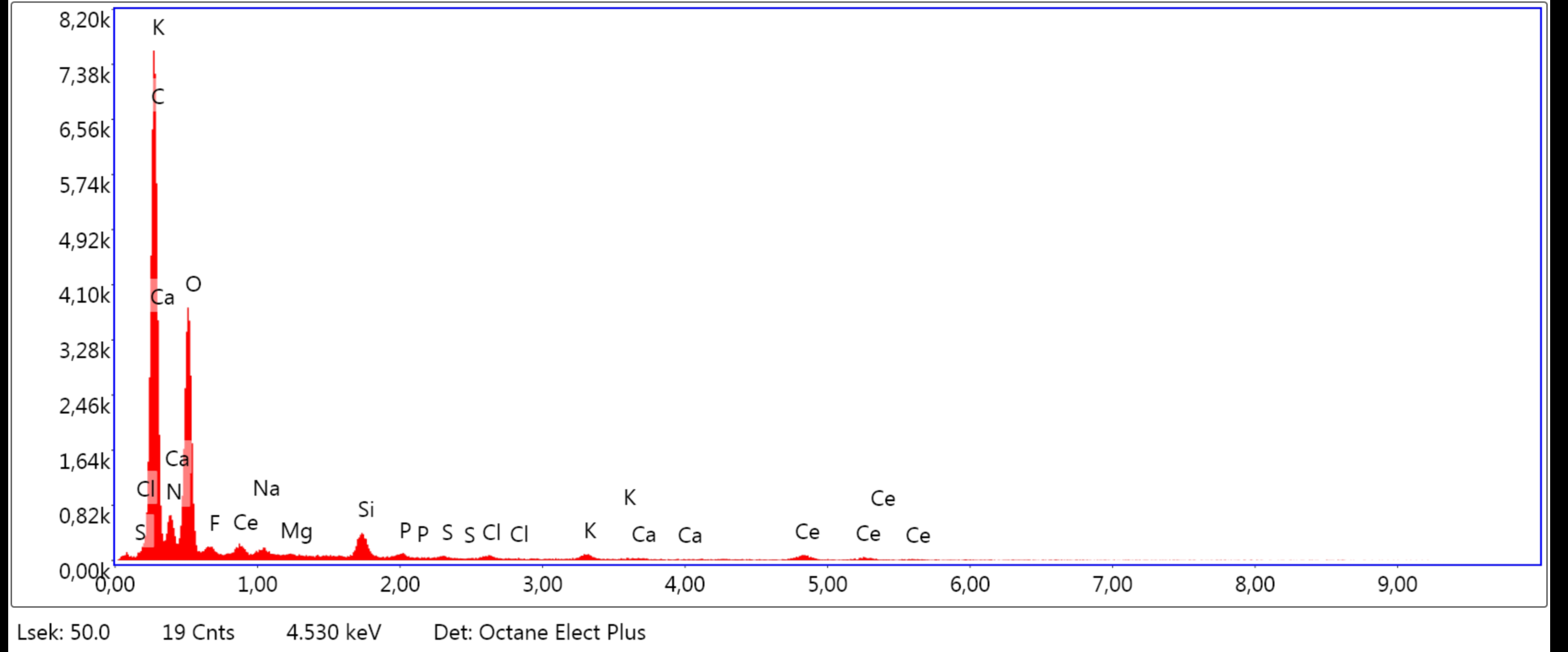


**Figure S2.** Exemplary EDS spectra of feed pellets loaded with CeO_2_ NM


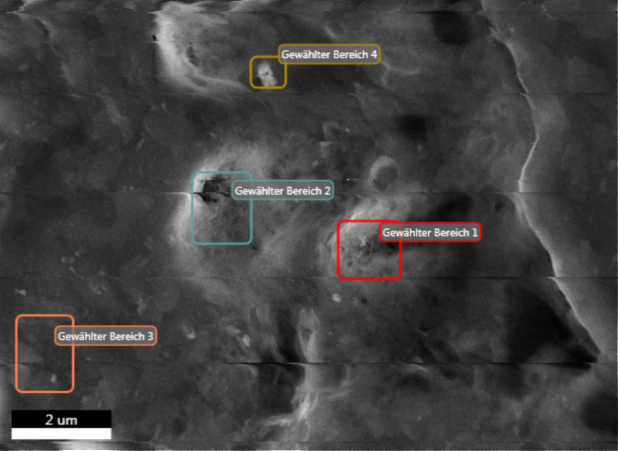


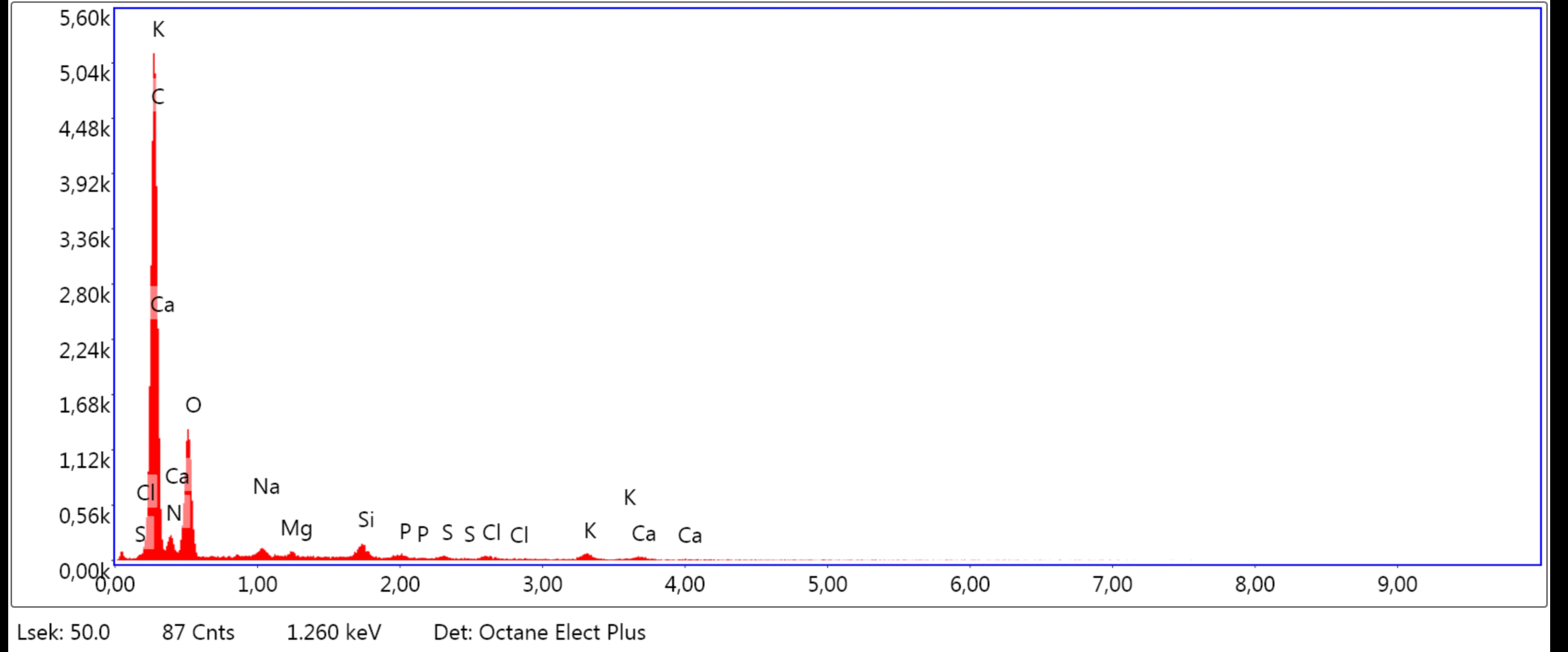


**Figure S3.** Exemplary EDS spectra of feed pellets loaded with SiO_2_ NM

^[1]^ Sofranko, A.; Wahle, T.; Heusinkveld, H. J.; Stahlmecke, B.; Dronov, M.; Pijnenburg, D.; Hilhorst, R.; Lamann, K.; Albrecht, C.; Schins, R. P. F., Neurotoxicology 2021, 84, 155-171.
